# General Mental Health State Indicators in Argentinean Women During Quarantine of up to 80-Day Duration for COVID-19 Pandemic

**DOI:** 10.3389/fgwh.2020.580652

**Published:** 2020-09-17

**Authors:** Lorena Cecilia López Steinmetz, Shao Bing Fong, Candela Abigail Leyes, María Agustina Dutto Florio, Juan Carlos Godoy

**Affiliations:** ^1^Laboratorio de Psicología, Instituto de Investigaciones Psicológicas (IIPsi), Universidad Nacional de Córdoba (UNC)—Consejo Nacional de Investigaciones Científicas y Técnicas (CONICET), Córdoba, Argentina; ^2^Decanato de Ciencias Sociales, Universidad Siglo 21, Córdoba, Argentina; ^3^University of Melbourne, Faculty of Science, Melbourne, VIC, Australia

**Keywords:** coronavirus, women's mental health, psychological distress, COVID-19, coping, social functioning, self-perceived health, quarantine

## Abstract

**Introduction:** Argentinean quarantine during the COVID-19 pandemic is one of the most long-lasting worldwide. We focused on the first 80-days of this quarantine on Argentinean women. Our aims were to analyze differences in general mental health state (MHS) indicators, by the (1) sites of residence with different prevalence of COVID-19 cases, and (2) quarantine duration; (3) to assess multiple relationships between each general MHS indicator and potentially affecting factors.

**Methods:** We used a cross-sectional design with convenience successive sampling (*N* = 5,013). The online survey included a socio-demographic questionnaire (elaborated *ad hoc*) with standardized and validated self-reported questionnaires (General Health Questionnaire, Kessler Psychological Distress Scale) measuring the MHS indicators: self-perceived health, psychological discomfort, social functioning and coping, and psychological distress.

**Results:** Worse self-perceived health and higher psychological discomfort affected significantly more women residing in sites with high prevalence of COVID-19 cases, compared to those residing in sites with intermediate prevalence, but effect sizes were small. Mean scores of all general MHS indicators were significantly worse for longer quarantine sub-periods (up to 53, 68, and 80-day duration) than for shorter sub-periods (up to seven, 13, and 25-day duration). Being a younger age, having mental disorder history, and longer quarantine durations were associated to worsening MHS, while the lack of previous suicide attempt has a protective effect.

**Discussion:** Our findings show that a worse MHS during quarantine may not be attributed to the objective risk of contagion (measured greater or less), and under quarantine, women MHS—as indicated by group central tendency measures—got worse as time went by. This strongly suggests that special attention needs to be paid to younger women and to women with history of mental disorder. Along with physical health, mental health must be a priority for the Government during and after quarantine and the COVID-19 pandemic.

## Introduction

The outbreak of the COVID-19 (coronavirus disease) started in Wuhan, China, on 31st December 2019. This virus promptly spread around the world, leading to the current COVID-19 pandemic declared on 11th March 2020 (1). On 1st August, 17,396,943 persons have become infected and 675,060 deaths have occurred globally due to the COVID-19 (2). On the same date, there were a total of 195,543 confirmed cases of COVID-19 and 3,596 fatal cases due to this disease in Argentina (3). In addition, there are a wide range of health concerns, beyond those physical effects directly attributable to the virus itself (4), which should be recognized in order to allow developing and implementing responses possible. Among such health concerns are mental health issues.

Due to the pandemic, hundreds of countries have adopted old-style sanitary measures—e.g., isolation, quarantine, social distancing, and community containment. As effective vaccine against COVID-19 is still unavailable, these measures play a critical role in containing the disease spread-rates (5). However, quarantine and social distancing-related measures due to the COVID-19 pandemic may produce undesirable mental health effects in the general population (6–8), such as anxiety, depressive symptoms, stress, and insomnia (8), among others. In addition, it is suspected that prolonged quarantine duration is likely to exacerbate these effects (6).

The negative mental health impacts of quarantine may vary by context and by groups. To the best of our knowledge, there are no studies focusing on the mental health effects of quarantine in women during the current pandemic of COVID-19, except for studies on particular women sub-groups, such as during pregnancy or within the first year after delivery (9, 10). Notwithstanding, women are one of the groups which may be particularly vulnerable to suffer from higher negative impacts on mental health from both the pandemic and the social distancing measures (11). Indeed, during the current pandemic, a nationwide survey among Chinese people showed that women had significantly higher psychological distress than their men counterparts (12). In view of all the aforementioned, research focusing on women mental health is a pressing request.

In Argentina, mandatory quarantine was established for all inhabitants—except for workers on essential services—since 20th March 2020 and for a duration of 2-weeks. Nonetheless, several quarantine extensions were afterward necessary. On 30th July, eight quarantine extensions had occurred, corresponding to a quarantine duration of 133-days and counting. Whether a negative mental health impact of quarantine is dependent on duration, lengthy Argentinean quarantines should indicate certain insights about it. In this paper, we focused on the first 80-days of this quarantine.

The aims of this research are 3-fold: to analyze differences in general mental health state (MHS) indicators (in terms of self-perceived health, psychological discomfort, social functioning and coping, and psychological distress), in Argentinean women, by (1) sites of residence with different prevalence of COVID-19 cases (per 100,000 inhabitants), and (2) quarantine duration; (3) to assess multiple relationships between each general MHS indicator and potentially affecting factors (age, sites of residence by prevalence of COVID-19 cases, mental disorder history, suicide attempt history, and quarantine duration) in the entire sample.

## Methods

### Sample and Procedure

This study used a cross-sectional design. Sampling was one of convenience, with successive samples, and included 5,013 Argentinean women from 18 years of age [M_age_ = 25.71, standard error [s.e.] ± 0.12; Median = 23; Range = 18–75], residing in one of the 23 Argentinean provinces, the Buenos Aires City (CABA) or momentarily stranded abroad due to travel bans and airport closures due the COVID-19 pandemic (Table S1). Data were collected since 17th March (i.e., 3 days before quarantine became mandatory in Argentina, but when quarantine was already strongly recommended by the Government to all Argentinean inhabitants) until 4th June 2020 (i.e., during mandatory Argentinean quarantine, up to the 5th quarantine extension announced by the Government, inclusive). Collection procedure was carried out via online, using Lime Survey software (UNC official license). This study was advertised in social networks with a brief mention to the general aim, general inclusion criteria (being women, Argentinean, being 18 years of age or older, currently reside in Argentina), and the link for the online survey. Upon accessing the survey, participants were initially presented with the information sheet and informed consent form approved by the Ethics Committee of the Institute of Psychological Research, Faculty of Psychology, National University of Córdoba (CEIIPsi-UNC-CONICET; comite.etica.iipsi@psicologia.unc.edu.ar).

### Instruments

*Sociodemographic questionnaire*. We developed a brief *ad hoc* questionnaire on sociodemographic data and other factors potentially affecting the current MHS. With this instrument we asked the participants about: age; current site of residence (options available between: each one of the 23 Argentinean provinces, the CABA or momentarily stranded abroad); mental disorder history (yes or no); suicide attempt history (yes, no, ideation); date (automatically recorded by the online survey system).

*General Health Questionnaire (GHQ-12)* (13). We used the Argentinean validation (Cronbach's alpha = 0.80) of the GHQ-12 (14). This is a 12-item measure, which evaluates the general dimension of *self-perceived health* and allows for discrimination in two sub-dimensions (6 items each): (a) unspecific psychological well-being/discomfort (hereinafter named as *psychological discomfort*), and (b) social functioning and coping. In the GHQ-12, the higher the score, the worse is the self-perceived health. In this research, we informed scores on the general dimension and on the two sub-dimensions. We used the dichotomous scoring (0-0-1-1), whose range of scores is between 0 and 12 for the entire scale and is between 0 and 6 for each sub-dimension. For this form of scoring, the cutoff scores for the entire scale indicating common mental disorders are 4 or 5 (13). We adopted the higher cutoff score (i.e., 5) for the entire scale. There are no cutoff scores for the sub-dimensions.

*Kessler Psychological Distress Scale (K-10)* (15). We used the Argentinean validation (Cronbach's alpha = 0.88) of the K-10 (16). This is a 10-item global dimensional measure of non-specific psychological distress (hereinafter named as *psychological distress*), which evaluates symptoms related to depression and anxiety, indicating the risk to suffer psychological distress but does not specify the disorder. The range of the K-10 scores is between 0 and 50, where a higher score indicates a higher psychological distress. This scale discriminates with precision between community cases and non-cases of DSM-IV disorders (17). Since there are no cutoff scores specific to the Argentinean population, we adopted the cutoff score of 20 (18) for deciding between cases and non-cases of any depressive and/or anxiety disorder. In addition, we used the following classification of the psychological distress: low (scores between 10 and 15), moderate (scores between 16 and 21), severe (scores between 22 and 29), and very severe (scores between 30 and 50) (19).

### Data Analysis

We performed all data analysis with RStudio version 3.6.2 (20). We considered *p* ≤ 0.05 as statistically significant. We report exact *p-*values, except for *p-*values under 0.001, where we report as < 0.001. Likewise, 95% confidence interval (CI) is informed when corresponded. Skewness and kurtosis were calculated in all indicators of general MHS, since it were in the range of acceptable values [−1 to +1 for skewness and −3 and +3 for kurtosis; (21)] (see Supplementary Material), parametric tests were applied.

For analyses corresponding to the first aim, we established the following categories of prevalence of COVID-19 confirmed cases per 100,000 inhabitants, based on official available data from 10th June (22): low (up to 10 confirmed cases per 100,000 inhabitants), intermediate (between 11 and 25 confirmed cases per 100,000 inhabitants), intermediate to high (between 26 and 50 confirmed cases per 100,000 inhabitants), and high (> 50 confirmed cases per 100,000 inhabitants). Then, we grouped the sites of residence into these categories of prevalence of COVID-19. However, each site of residence corresponded to one of three of these categories, but none corresponded to the intermediate to high category (Table S2). Thus, prevalence categories grouping all our samples were as follow: *low* (*n* = 952 participants from the provinces of Jujuy, Salta, Tucumán, Santiago del Estero, Formosa, Misiones, Entre Ríos, Catamarca, San Juan, San Luis, La Pampa, and Chubut), *intermediate* (*n* = 1,938 participants from the provinces of Corrientes, Santa Fe, Mendoza, La Rioja, Córdoba, Neuquén, and Santa Cruz), and *high* (*n* = 2,123 participants from the provinces of Buenos Aires, CABA, Chaco, Río Negro, Tierra del Fuego, and currently stranded abroad). As stated in the first aim, we analyzed differences in each general MHS indicator (i.e., self-perceived health, psychological discomfort, social functioning and coping, and psychological distress) by the sites of residence with different prevalence of COVID-19 cases. Additionally, we explored differences in age, in proportions of mental disorder history (presence), and in proportions of suicide attempt history (presence, absence, ideation) by the sites of residence with different prevalence of COVID-19 cases.

For addressing the second aim, we divided the entire sample into six groups according to the sub-periods of quarantine duration: (a) participants answering during 17–23 March 2020, i.e., first week of data collection before the quarantine extension, named as first *week pre-quarantine extension* (*n* = 1,490) and corresponding to a quarantine duration of up to 7-days; (b) participants answering during 24–29 March 2020, named as second *week pre-quarantine extension* (*n* = 495) and corresponding to a quarantine duration of up to 13-days; (c) participants answering during 30 March-10 April 2020, i.e., sub-period after the first quarantine extension, named as first *extension* (*n* = 766) and corresponding to a quarantine duration of up to 25-days; (d) participants answering during 11 April-08 May 2020, i.e., sub-period after the second quarantine extension and including the third extension, named as *second/third extensions* (*n* = 594) and corresponding to a quarantine duration of up to 53-days; (e) participants answering during 09–23 May 2020, i.e., sub-period after the fourth quarantine extension, named as *fourth extension* (*n* = 652) and corresponding to a quarantine duration of up to 68-days; (f) participants answering during 24 May-04 June 2020, i.e., sub-period after the fifth quarantine extension, named as fifth *extension* (*n* = 1,016) and corresponding to a quarantine duration of up to 80-days. As stated in the second aim, we analyzed differences in each general MHS indicator (i.e., self-perceived health, psychological discomfort, social functioning and coping, and psychological distress) by quarantine duration. Additionally, we explored differences in age, in proportions of mental disorder history (presence), and in proportions of suicide attempt history (presence, absence, ideation) by quarantine duration.

For addressing the first and second aims of this research, we applied one-way between-groups ANOVA (when the criterion variable was numerical) or test for equality of proportions (when the criterion variable was categorical). When significant differences were found, Tukey's *post hoc* test or pairwise comparisons of proportions (Test of equal or given proportions with two-sided alternative hypothesis) were applied, correspondingly.

For addressing the third aim, we run multiple linear regressions rather than ANCOVA because the emphasis of this aim was on the dependent outcome variable. We considered the following potentially affecting factors: age, sites of residence by prevalence of COVID-19 cases, mental disorder history, suicide attempt history, and quarantine duration. We considered these based on both the literature (6) and findings from a previous analysis we have carried out in another sample of Argentinean population of both sexes (López Steinmetz et al. under review). Prior to running regression analyses, we assessed multicollinearity by using the variance inflation factor (VIF), the mean VIF, and the tolerance statistics, and we adopted the following criteria for interpreting these outcomes: a) if the largest VIF is > 10, then there is cause of concern, b) if the average VIF is substantially > 1, then the regression may be biased, c) tolerance below 0.1 and below 0.2 indicates a serious and a potential problem, respectively (23). For the initial model, the VIF values were all well below 10, the tolerance statistics were all well above 0.2, and the average VIF was close to 1 (Table S3), indicating that there was no collinearity within our data. Thus, for each general MHS indicator (i.e., self-perceived health, psychological discomfort, social functioning and coping, and psychological distress), we tested (with the *lm* function) a starting model including all the predictors mentioned above (i.e, age, sites of residence by prevalence of COVID-19 cases, mental disorder history, suicide attempt history, and quarantine duration) for the entire sample. We used a stepwise method of regression where decisions about the order in which predictors are entered into the model are based on a purely mathematical criterion (23). Specifically, for each MHS indicator, we performed a stepwise model selection in both directions (i.e., forward and backward) by using the exact Akaike's Information Criterion (AIC). To do this, we used the *stepAIC* function from the *MASS* package. This function performs stepwise model selection by using the exact AIC to compare fitted models, where the smaller the AIC indicates a better fit. For each general MHS indicator, we tried only additive models. For the model best fitting each MHS indicator, we reported 95% confidence interval (CI), the coefficient of determination (*r*^2^), and the adjusted R-squared (adj *r*^2^). Likewise, for providing a measure of error prediction, we calculated the error rate by dividing the residual standard error (RSE) by the mean outcome variable.

For analyses corresponding to the three aims, we computed effect sizes (ES) by using the *effectsize::cohens_f* function from the *sjstats* package. We adopted the Cohen's effect size conventions, for one-way ANOVA: *f* = 0.10 small, *f* = 0.25 medium, and *f* = 0.40 large; for multiple regression: *f* = 0.02 small, *f* = 0.15 medium, and *f* = 0.35 large.

## Results

### Differences in General Mental Health State by Sites of Residence With Different Prevalence of COVID-19

Regarding general MHS by sites of residence with different prevalence of COVID-19 cases, statistically significant differences were found in self-perceived health [F_(2)_ = 5.15, *p* = 0.006; ES = 0.05, 90% CI: 0.02–0.07] and in psychological discomfort [F_(2)_ = 5.66, *p* = 0.003; ES = 0.05, 90% CI: 0.02–0.07]. In both MHS indicators, these differences were observed between the high and the intermediate prevalence of COVID-19 cases. In self-perceived health, differences were also meaningful between the low and the intermediate level of prevalence of COVID-19 cases (Table 1). In all sites, the mean scores of self-perceived health were above the cutoff score indicating common mental disorders (Table 2). In addition, a significant difference was found in social functioning and coping by sites of residence with different prevalence of COVID-19 cases [F_(2)_ = 3.17, *p* = 0.04; ES = 0.04, 90% CI: 0.00–0.06], but this difference does not remain significant in the *post hoc* test (Table 1). Likewise, no significant differences were found in psychological distress [F_(2)_ = 0.35, *p* = 0.71; ES = 0.01, 90% CI: 0.00–0.03; Table 1]. In all sites, the mean scores of psychological distress were above the cutoff score for deciding between cases and non-cases of any depressive and/or anxiety disorder; mean scores in all sites indicated severe psychological distress (Table 2).

**Table 1:** Multiple comparisons of means^a^ in general mental health state (MHS) scores and mean age by sites of residence with different prevalence of COVID-19 cases.

**MHS indicators and age**	**Sites of residence by prevalence levels of COVID-19 cases^b^**	**Dif**	**95% CI**		***p* adj^c^**
			**Lower**	**Upper**	
Self-perceived health	Intermediate-High	−0.32	−0.58	−0.07	**0.008**
	Low-High	−0.002	−0.32	0.31	1.00
	Low-Intermediate	0.32	−0.0004	0.64	**0.05**
Psychological discomfort	Intermediate-High	−0.19	−0.33	−0.05	**0.003**
	Low-High	−0.03	−0.20	0.14	0.90
	Low-Intermediate	0.16	−0.01	0.33	0.08
Social functioning and coping	Intermediate-High	−0.13	−0.28	0.01	0.08
	Low-High	0.03	−0.15	0.21	0.92
	Low-Intermediate	0.16	−0.02	0.34	0.09
Psychological distress	Intermediate-High	−0.21	−0.82	0.40	0.69
	Low-High	−0.06	−0.81	0.70	0.98
	Low-Intermediate	0.16	−0.61	0.92	0.88
Age	Intermediate-High	0.43	−0.21	1.08	0.25
	Low-High	0.89	0.09	1.69	**0.02**
	Low-Intermediate	0.46	−0.35	1.27	0.38

*Dif, Difference; 95% CI, 95% Confidence Interval; Lower—Upper, Lower and upper limits of 95% confidence intervals; p adj, Adjusted p-value; Low, up to 10 confirmed cases of COVID-19 per 100,000 inhabitants; Intermediate, between 11 and 25 confirmed cases of COVID-19 per 100,000 inhabitants; High, > 50 confirmed cases of COVID-19 per 100,000 inhabitants*.

a *Multiple comparisons of means were carried out with Tukey post hoc test*.

b *Categories based on available official data published by the Argentinean Government on 10th June 2020 (22): low (up to 10 confirmed cases per 100,000 inhabitants), intermediate (between 11 and 25 confirmed cases per 100,000 inhabitants), intermediate to high (between 26 and 50 confirmed cases per 100,000 inhabitants), and high (> 50 confirmed cases per 100,000 inhabitants). No sites corresponded to the intermediate to high category of prevalence*.

c *Exact p-values are informed, except for p-values under 0.001, which are informed as <0.001. Statistically significant p-values are highlighted in bold*.

**Table 2:** Mental health state, age, mental disorder history, and suicide attempt history by sites of residence with different prevalence of COVID-19 cases.

	**Sites of residence by prevalence of COVID-19 cases** ^b^		
**Mental health-related variables^a^**	**Low (*n =* 952)**	**Intermediate (*n =* 1,938)**	**High (*n =* 2,123)**
Self-perceived health	5.83 (± 0.11)	5.51 (± 0.08)	5.83 (± 0.07)
Psychological discomfort	3.43 (± 0.06)	3.27 (± 0.04)	3.46 (± 0.04)
Social functioning and coping	2.40 (± 0.06)	2.24 (± 0.04)	2.37 (± 0.04)
Psychological distress	26.40 (± 0.27)	26.25 (± 0.19)	26.46 (± 0.18)
Age	26.27 (± 0.27)	25.81 (± 0.21)	25.38 (± 0.18)
Mental disorder history (presence or absence)	25.73% presence, 74.26% absence	27.29% presence, 72.70% absence	26.80% presence, 73.20% absence
Suicide attempt history (presence, absence, ideation)	9.24% presence, 55.25% absence, 35.50% ideation	8.87% presence, 57.38% absence, 33.75% ideation	7.39% presence, 57.18% absence, 35.42% ideation

*Low, up to 10 confirmed cases per 100,000 inhabitants; Intermediate, between 11 and 25 confirmed cases per 100,000 inhabitants; Intermediate to high, between 26 and 50 confirmed cases per 100,000 inhabitants; High, > 50 confirmed cases per 100,000 inhabitants*.

a *For the variables self-perceived health, psychological discomfort, social functioning and coping, psychological distress, and age, mean and standard error are informed, while for mental disorder history and suicide attempt history, distributions by percentages are informed*.

b *Categories based on available official data published by the Argentinean Government on 10th June 2020 (22). No sites corresponded to the intermediate to high category of prevalence*.

The age of participants significantly differed between sites of residence with different prevalence of COVID-19 cases [F_(2)_ = 3.64, *p* = 0.03], although with a small effect size (ES = 0.04, 90% CI: 0.01–0.06). However, the difference was only meaningful between sites with low and high prevalence of COVID-19 cases, but not between sites with low and intermediate prevalence, nor between intermediate and high prevalence of COVID-19 cases (Table 1). Likewise, by sites of residence with low, intermediate, and high prevalence of COVID-19 cases, no differences were found in proportions of participants having mental disorder history [X-squared_(2)_ = 0.79, *p* = 0.67] nor in proportions of participants with suicide attempt history [X-squared_(2)_ = 4.23, *p* = 0.12], without suicide attempt history [X-squared_(2)_ = 1.30, *p* = 0.52] or with suicidal ideation history [X-squared_(2)_ = 1.52, *p* = 0.47]. Mean age of participants and proportions of participants having mental disorder history and suicide attempt history by sites of residence are shown in Table 2.

### Differences in General Mental Health State by Quarantine Sub-periods

**Figure 1:**
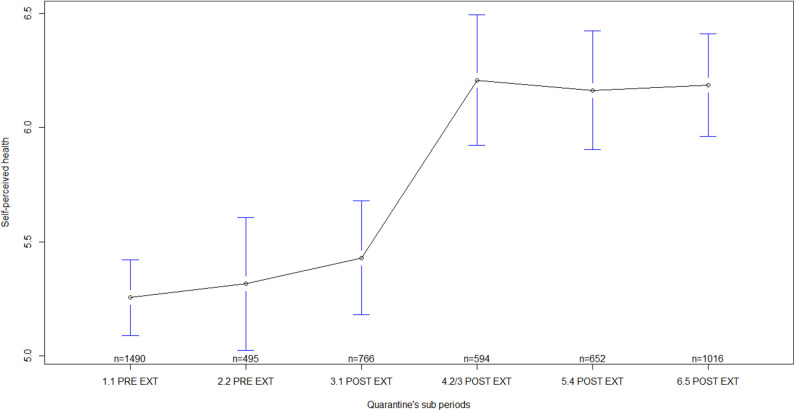
Self-perceived health by quarantine sub-periods. Mean plot with 95% confidence interval. Self-perceived health as measured by the General Health Questionnaire (scores from the entire scale), in which higher scores indicate worse self-perceived health. 1.1 PRE EXT = First week of quarantine before extension, including participants answering during 17–23 March 2020; 2.2 PRE EXT = Second week of quarantine before extension, including participants answering during 24–29 March 2020; 3.1 POST EXT = Sub-period after the first quarantine extension, including participants answering during 30 March to 10 April 2020; 4.2/3 POST EXT = Sub-period after the second quarantine extension and including the third extension, with participants answering during 11 April to 08 May 2020; 5.4 POST EXT = Sub-period after the fourth quarantine extension, including participants answering during 09–23 May 2020; 6.5 POST EXT = Sub-period after the fifth quarantine extension, including participants answering during 24 May to 04 June 2020.

**Figure 2:**
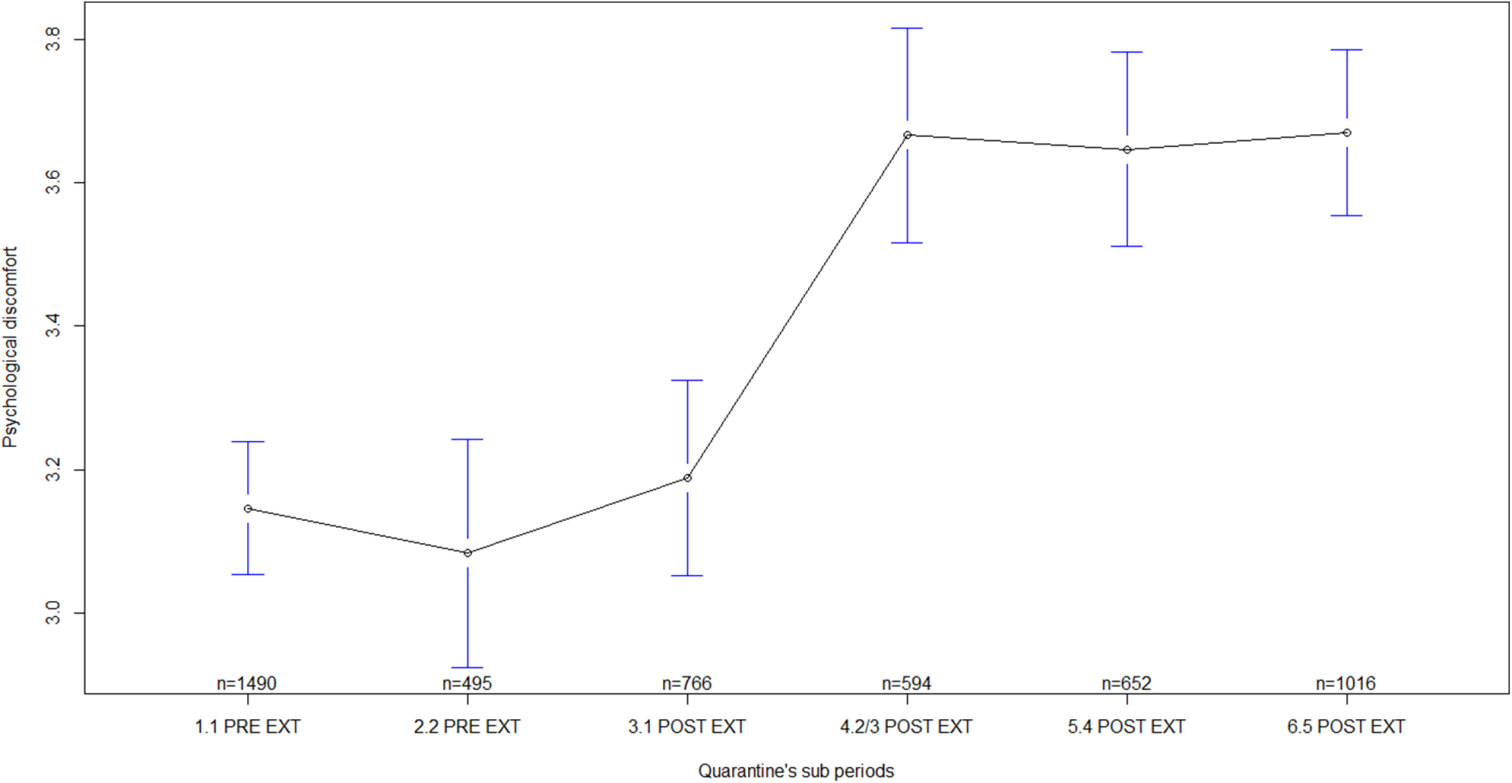
Psychological discomfort by quarantine sub-periods. Mean plot with 95% confidence interval. Psychological discomfort as measured by the General Health Questionnaire (scores from the sub-scale measuring unspecific psychological well-being/discomfort), in which higher scores indicate higher psychological discomfort. 1.1 PRE EXT = First week of quarantine before extension, including participants answering during 17–23 March 2020; 2.2 PRE EXT = Second week of quarantine before extension, including participants answering during 24–29 March 2020; 3.1 POST EXT = Sub-period after the first quarantine extension, including participants answering during 30 March to 10 April 2020; 4.2/3 POST EXT = Sub-period after the second quarantine extension and including the third extension, with participants answering during 11 April to 08 May 2020; 5.4 POST EXT = Sub-period after the fourth quarantine extension, including participants answering during 09–23 May 2020; 6.5 POST EXT = Sub-period after the fifth quarantine extension, including participants answering during 24 May to 04 June 2020.

**Figure 3:**
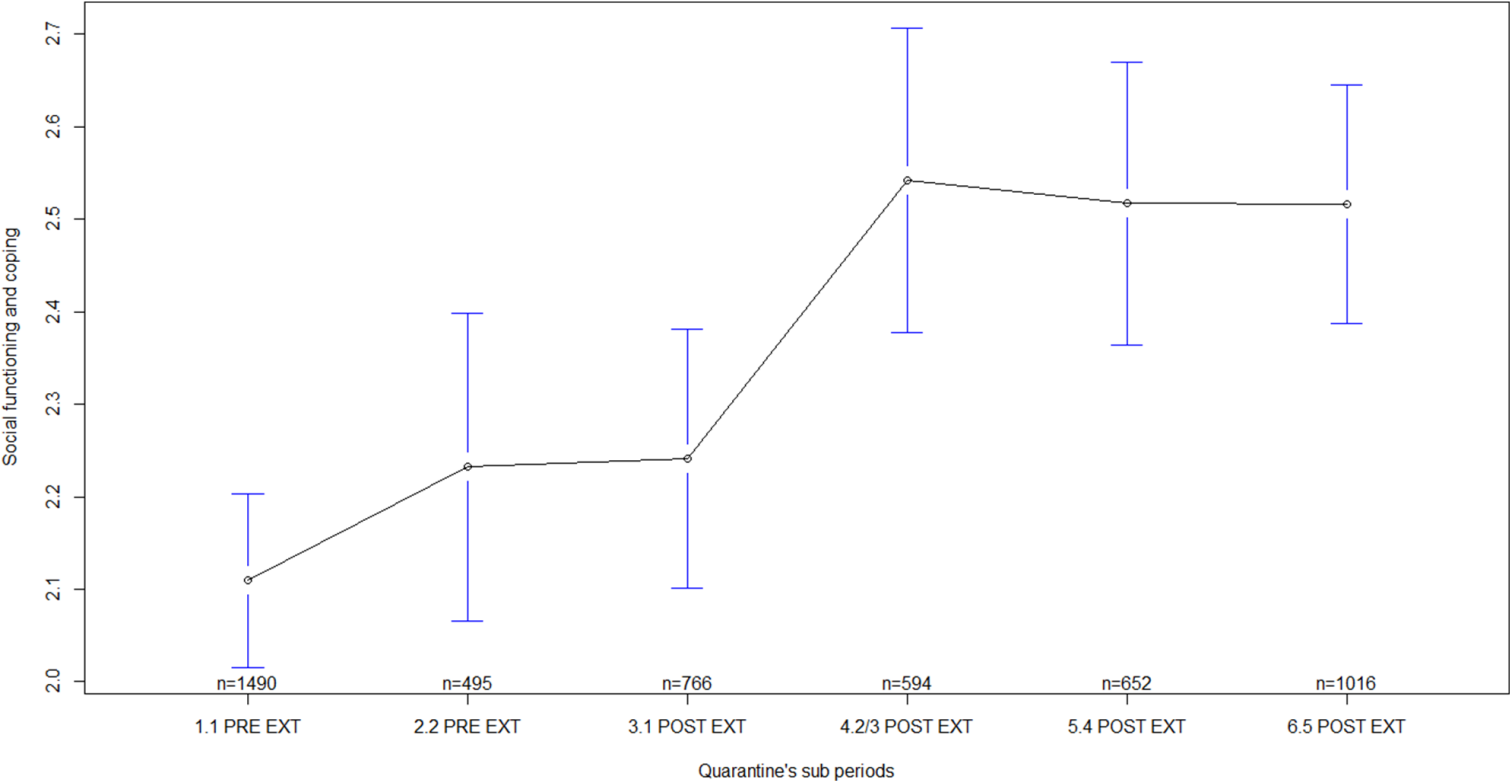
Social functioning and coping by quarantine sub-periods. Mean plot with 95% confidence interval. Social functioning and coping as measured by the General Health Questionnaire (scores from the sub-scale measuring social functioning and coping), in which higher scores indicate worse social functioning and coping. 1.1 PRE EXT = First week of quarantine before extension, including participants answering during 17–23 March 2020; 2.2 PRE EXT = Second week of quarantine before extension, including participants answering during 24–29 March 2020; 3.1 POST EXT = Sub-period after the first quarantine extension, including participants answering during 30 March to 10 April 2020; 4.2/3 POST EXT = Sub-period after the second quarantine extension and including the third extension, with participants answering during 11 April to 08 May 2020; 5.4 POST EXT = Sub-period after the fourth quarantine extension, including participants answering during 09–23 May 2020; 6.5 POST EXT = Sub-period after the fifth quarantine extension, including participants answering during 24 May to 04 June 2020.

**Figure 4:**
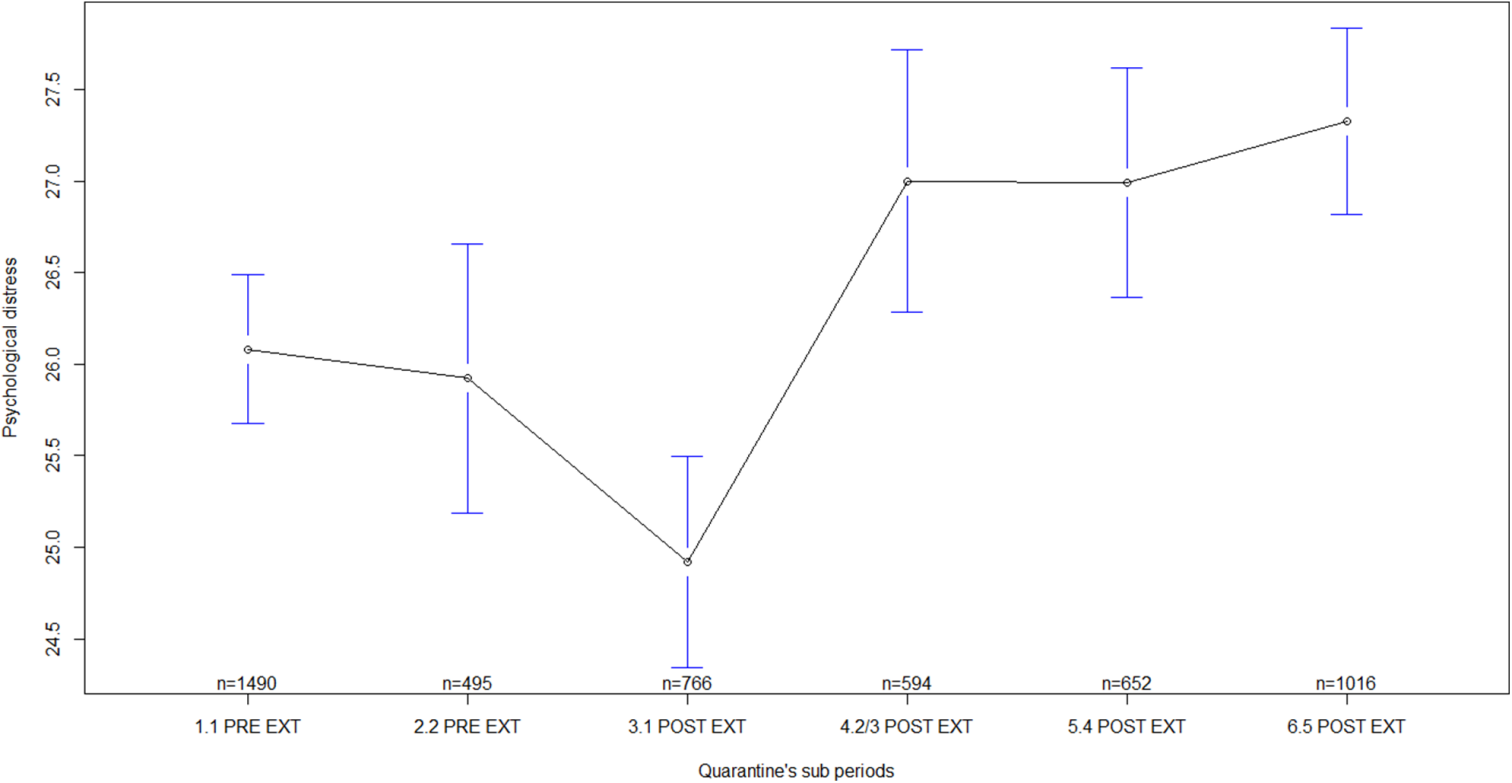
Psychological distress by quarantine sub-periods. Mean plot with 95% confidence interval. Psychological distress as measured by the Kessler Psychological Distress Scale, in which higher scores indicate higher psychological distress. 1.1 PRE EXT = First week of quarantine before extension, including participants answering during 17–23 March 2020; 2.2 PRE EXT = Second week of quarantine before extension, including participants answering during 24–29 March 2020; 3.1 POST EXT = Sub-period after the first quarantine extension, including participants answering during 30 March to 10 April 2020; 4.2/3 POST EXT = Sub-period after the second quarantine extension and including the third extension, with participants answering during 11 April to 08 May 2020; 5.4 POST EXT = Sub-period after the fourth quarantine extension, including participants answering during 09–23 May 2020; 6.5 POST EXT = Sub-period after the fifth quarantine extension, including participants answering during 24 May to 04 June 2020.

Regarding general MHS by quarantine sub-periods, statistically significant differences were found in all the indicators measured, i.e., in self-perceived health [F_(5)_ = 16.18, *p* < 0.001; ES = 0.13, 90% CI: 0.10–0.15], in psychological discomfort [F_(5)_ = 19.69, *p* < 0.001; ES = 0.14, 90% CI: 0.11–0.16], in social functioning and coping [F_(5)_ = 8.69, *p* < 0.001; ES = 0.09, 90% CI: 0.06–0.11], and in psychological distress [F_(5)_ = 9.59, *p* < 0.001; ES = 0.10, 90% CI: 0.07–0.12]. Several differences were observed between sub-periods before quarantine extensions and sub-periods after quarantine extensions (Table 3). In general, mean scores of MHS during sub-periods before quarantine extensions were lower than the mean scores after the extensions, mainly, during sub-periods corresponding to the second/third, fourth, and fifth extensions of quarantine (Table 4; Figures 1–4). In all of the quarantine sub-periods, mean scores of self-perceived health were above the cutoff score indicating common mental disorders. Likewise, in all of the sub-periods, mean scores of psychological distress were above the cutoff score for deciding between cases and non-cases of any depressive and/or anxiety disorder. For all sub-periods, mean scores indicated severe psychological distress (Table 4).

**Table 3:** Multiple comparisons^a^ of means in general mental health state (MHS) scores and age by quarantine sub-periods.

**MHS indicators and age**	**Quarantine sub-periods**	**Dif**	**95% CI**		***p* adj^b^**
			**Lower**	**Upper**	
Self-perceived health	1. First week pre-extension–2. Second week pre-extension	0.06	−0.45	0.57	1.00
	1. First week pre-extension–3. First extension	0.17	−0.26	0.61	0.86
	1. First week pre-extension–4. Second/third extensions	0.95	0.48	1.43	**< 0.001**
	1. First week pre-extension–5. Fourth extension	0.91	0.45	1.37	**< 0.001**
	1. First week pre-extension–6. Fifth extension	0.93	0.53	1.33	**< 0.001**
	2. Second week pre-extension–3. First extension	0.11	−0.45	0.68	0.99
	2. Second week pre-extension–4. Second/third extensions	0.89	0.30	1.49	**< 0.001**
	2. Second week pre-extension–5. Fourth extension	0.85	0.26	1.43	**< 0.001**
	2. Second week pre-extension–6. Fifth extension	0.87	0.33	1.41	**< 0.001**
	3. First extension–4. Second/third extensions	0.78	0.24	1.32	**< 0.001**
	3. First extension–5. Fourth extension	0.73	0.21	1.26	**0.001**
	3. First extension–6. Fifth extension	0.76	0.29	1.23	**< 0.001**
	4. Second/third extensions–5. Fourth extension	−0.04	−0.60	0.51	1.00
	4. Second/third extensions–6. Fifth extension	−0.02	−0.53	0.48	1.00
	5. Fourth extension–6. Fifth extension	0.02	−0.47	0.51	1.00
Psychological discomfort	1. First week pre-extension–2. Second week pre-extension	−0.06	−0.33	0.21	0.99
	1. First week pre-extension–3. First extension	0.04	−0.19	0.28	0.99
	1. First week pre-extension–4. Second/third extensions	0.52	0.27	0.78	**< 0.001**
	1. First week pre-extension–5. Fourth extension	0.50	0.25	0.75	**< 0.001**
	1. First week pre-extension–6. Fifth extension	0.52	0.31	0.74	**< 0.001**
	2. Second week pre-extension–3. First extension	0.10	−0.20	0.41	0.92
	2. Second week pre-extension–4. Second/third extensions	0.58	0.26	0.90	**< 0.001**
	2. Second week pre-extension–5. Fourth extension	0.56	0.25	0.88	**< 0.001**
	2. Second week pre-extension–6. Fifth extension	0.59	0.30	0.88	**< 0.001**
	3. First extension–4. Second/third extensions	0.48	0.19	0.77	**< 0.001**
	3. First extension–5. Fourth extension	0.46	0.18	0.74	**< 0.001**
	3. First extension–6. Fifth extension	0.48	0.23	0.73	**< 0.001**
	4. Second/third extensions–5. Fourth extension	−0.02	−0.32	0.28	1.00
	4. Second/third extensions–6. Fifth extension	0.004	−0.27	0.27	1.00
	5. Fourth extension–6. Fifth extension	0.02	−0.24	0.29	1.00
Social functioning and coping	1. First week pre-extension–2. Second week pre-extension	0.12	−0.17	0.41	0.83
	1. First week pre-extension–3. First extension	0.13	−0.12	0.38	0.66
	1. First week pre-extension–4. Second/third extensions	0.43	0.16	0.70	**< 0.001**
	1. First week pre-extension–5. Fourth extension	0.41	0.14	0.67	**< 0.001**
	1. First week pre-extension–6. Fifth extension	0.41	0.18	0.63	**< 0.001**
	2. Second week pre-extension–3. First extension	0.01	−0.31	0.33	1.00
	2. Second week pre-extension–4. Second/third extensions	0.31	−0.03	0.65	0.10
	2. Second week pre-extension–5. Fourth extension	0.28	−0.05	0.62	0.15
	2. Second week pre-extension–6. Fifth extension	0.28	−0.02	0.59	0.10
	3. First extension–4. Second/third extensions	0.30	−0.01	0.61	0.06
	3. First extension–5. Fourth extension	0.27	−0.02	0.57	0.10
	3. First extension–6. Fifth extension	0.27	0.01	0.54	**0.04**
	4. Second/third extensions–5. Fourth extension	−0.02	−0.34	0.29	1.00
	4. Second/third extensions–6. Fifth extension	−0.03	−0.32	0.26	1.00
	5. Fourth extension–6. Fifth extension	−0.001	−0.28	0.28	1.00
Psychological distress	1. First week pre-extension–2. Second week pre-extension	−0.16	−1.38	1.06	1.00
	1. First week pre-extension–3. First extension	−1.16	−2.21	−0.12	**0.02**
	1. First week pre-extension–4. Second/third extensions	0.92	−0.22	2.06	0.20
	1. First week pre-extension–5. Fourth extension	0.91	−0.19	2.01	0.17
	1. First week pre-extension–6. Fifth extension	1.24	0.29	2.20	**0.003**
	2. Second week pre-extension–3. First extension	−1.00	−2.36	0.35	0.28
	2. Second week pre-extension–4. Second/third extensions	1.08	−0.35	2.51	0.26
	2. Second week pre-extension–5. Fourth extension	1.07	−0.33	2.47	0.25
	2. Second week pre-extension–6. Fifth extension	1.40	0.12	2.69	**0.02**
	3. First extension–4. Second/third extensions	2.08	0.80	3.37	**< 0.001**
	3. First extension–5. Fourth extension	2.07	0.82	3.33	**< 0.001**
	3. First extension–6. Fifth extension	2.41	1.29	3.54	**< 0.001**
	4. Second/third extensions–5. Fourth extension	−0.01	−1.34	1.32	1.00
	4. Second/third extensions–6. Fifth extension	0.33	−0.89	1.54	0.97
	5. Fourth extension–6. Fifth extension	0.34	−0.84	1.52	0.96
Age	1. First week pre-extension–2. Second week pre-extension	0.93	−0.30	2.17	0.26
	1. First week pre-extension–3. First extension	5.28	4.22	6.34	**< 0.001**
	1. First week pre-extension–4. Second/third extensions	5.80	4.65	6.96	**< 0.001**
	1. First week pre-extension–5. Fourth extension	4.02	2.90	5.14	**< 0.001**
	1. First week pre-extension–6. Fifth extension	5.61	4.64	6.58	**< 0.001**
	2. Second week pre-extension–3. First extension	4.34	2.97	5.72	**< 0.001**
	2. Second week pre-extension–4. Second/third extensions	4.87	3.42	6.32	**< 0.001**
	2. Second week pre-extension–5. Fourth extension	3.08	1.66	4.50	**< 0.001**
	2. Second week pre-extension–6. Fifth extension	4.67	3.37	5.98	**< 0.001**
	3. First extension–4. Second/third extensions	0.53	−0.78	1.83	0.86
	3. First extension–5. Fourth extension	−1.26	−2.53	0.01	0.05
	3. First extension–6. Fifth extension	0.33	−0.81	1.47	0.96
	4. Second/third extensions–5. Fourth extension	−1.79	−3.14	−0.44	**0.002**
	4. Second/third extensions–6. Fifth extension	−0.20	−1.43	1.03	0.99
	5. Fourth extension–6. Fifth extension	1.59	0.39	2.79	**0.002**

*Dif, Difference; 95% CI, 95% Confidence Interval; Lower—Upper, Lower and upper limits of 95% confidence intervals; p adj, Adjusted p-value; Low, up to 10 confirmed cases of COVID-19 per 100,000 inhabitants; Intermediate, between 11 and 25 confirmed cases of COVID-19 per 100,000 inhabitants; High, > 50 confirmed cases of COVID-19 per 100,000 inhabitants. 1. First week pre-extension = First week of quarantine before extension, including participants answering during 17–23 March 2020; 2. Second week pre-extension = Second week of quarantine before extension, including participants answering during 24–29 March 2020; 3. First extension = Sub-period after the first quarantine extension, including participants answering during 30 March to 10 April 2020; 4. Second/third extensions = Sub-period after the second quarantine extension and including the third extension, with participants answering during 11 April to 08 May 2020; 5. Fourth extension = Sub-period after the fourth quarantine extension, including participants answering during 09–23 May 2020; 6. Fifth extension = Sub-period after the fifth quarantine extension, including participants answering during 24 May to 04 June 2020*.

a *Multiple comparisons of means were carried out with Tukey post hoc test*.

b *Exact p-values are informed, except for p-values under 0.001, which are informed as <0.001. Statistically significant p-values are highlighted in bold*.

**Table 4:** Mental health state, age, mental disorder history, and suicide attempt history by quarantine sub-periods.

	**Quarantine sub-periods**					
**Mental health-related variables^a^**	**1. First week pre-extension (*n =* 1,490)**	**2. Second week pre-extension (*n =* 495)**	**3. First extension (*n =* 766)**	**4. Second/third extensions (*n =* 594)**	**5. Fourth extension (*n =* 652)**	**6. Fifth extension (*n =* 1,016)**
Self-perceived health	5.25 (± 0.08)	5.31 (± 0.15)	5.43 (±0.13)	6.21 (± 0.15)	6.16 (± 0.13)	6.19 (± 0.11)
Psychological discomfort	3.15 (± 0.05)	3.08 (± 0.08)	3.19 (± 0.07)	3.67 (± 0.08)	3.65 (± 0.07)	3.67 (± 0.06)
Social functioning and coping	2.11 (± 0.05)	2.23 (± 0.08)	2.24 (± 0.07)	2.54 (± 0.08)	2.52 (± 0.08)	2.51 (± 0.06)
Psychological distress	26.08 (± 0.21)	25.92 (± 0.37)	24.92 (± 0.29)	27.00 (± 0.36)	27.00 (± 0.32)	27.33 (± 0.26)
Age	22.47 (± 0.10)	23.40 (± 0.24)	27.74 (± 0.38)	28.27 (± 0.40)	26.48 (± 0.37)	28.08 (± 0.33)
Mental disorder history (presence or absence)	25.91% presence, 74.09% absence	24.04% presence, 75.96% absence	26.76% presence, 73.24% absence	27.44% presence, 72.56% absence	25.46% presence, 74.54% absence	29.92% presence, 70.08% absence
Suicide attempt history (presence, absence, ideation)	7.92% presence, 51.54% absence, 40.54% ideation	9.29% presence, 53.53% absence, 37.17% ideation	8.49% presence, 59.79% absence, 31.72% ideation	9.76% presence, 58.08% absence, 32.15% ideation	7.21% presence, 60.74% absence, 32.05% ideation	8.17% presence, 61.02% absence, 30.81% ideation

*1. First week pre-extension = First week of quarantine before extension, including participants answering during 17–23 March 2020; 2. Second week pre-extension = Second week of quarantine before extension, including participants answering during 24–29 March 2020; 3. First extension = Sub-period after the first quarantine extension, including participants answering during 30 March to 10 April 2020; 4. Second/third extensions = Sub-period after the second quarantine extension and including the third extension, with participants answering during 11 April to 08 May 2020; 5. Fourth extension = Sub-period after the fourth quarantine extension, including participants answering during 09–23 May 2020; 6. Fifth extension = Sub-period after the fifth quarantine extension, including participants answering during 24 May to 04 June 2020*.

a *For the variables self-perceived health, psychological discomfort, social functioning and coping, psychological distress, and age, mean, and standard error are informed, while for mental disorder history, and suicide attempt history, percentages are informed*.

The age of participants significantly differed by quarantine sub-periods [F_(2)_ = 89.90, *p* < 0.001], with a medium effect size (ES = 0.30, 90% CI: 0.27–0.32) (Table 3). In general, the mean age of the sub-group of participants was higher for all quarantine extensions (i.e., four of the six sub-periods analyzed) compared to quarantine prior extensions (Table 4). On the other hand, there were no significant differences in participants with mental disorder history [X-squared_(5)_ = 8.30, *p* = 0.14] nor with suicide attempt history [X-squared_(5)_ = 3.67, *p* = 0.60] by quarantine sub-periods. However, there were significant differences in participants without suicide attempt history [X-squared_(5)_ = 33.62, *p* < 0.001] and with suicidal ideation history [X-squared_(5)_ = 37.18, *p* < 0.001] by quarantine sub-periods. For the absence of suicide attempt history, these differences were only meaningful between the first week of pre-quarantine extension (lower proportions of participants without suicide attempt history) and the first, fourth, and fifth extensions (higher proportions of participants without suicide attempt history). For suicidal ideation history, these differences were only meaningful between the first week pre-quarantine extension (higher proportions of participants with suicidal ideation history) and the first, second/third, fourth, and fifth extensions (lower proportions of participants with suicidal ideation history). Results on pairwise comparisons of proportions in participants both without suicide attempt history and with suicidal ideation history are shown in Table 5. Proportions of participants having mental disorder history and suicide attempt history by quarantine sub-periods are shown in Table 4.

**Table 5:** Pairwise comparisons of proportions in participants without suicide attempt history (lower triangle) and with suicidal ideation history (upper triangle) by quarantine sub-periods.

	**Pairwise comparisons of proportions^a^ (** ***p-*** **values^b^)**					
**Quarantine sub-periods**	**1**.	**2**.	**3**.	**4**.	**5**.	**6**.
1. First week pre-extension	–	1.00	**0.001**	**0.005**	**0.003**	**< 0.001**
2. Second week pre-extension	1.00	–	0.53	0.76	0.73	0.17
3. First extension	**0.003**	0.29	–	1.00	1.00	1.00
4. Second/third extensions	0.09	1.00	1.00	–	1.00	1.00
5. Fourth extension	**0.001**	0.17	1.00	1.00	–	1.00
6. Fifth extension	**< 0.001**	0.08	1.00	1.00	1.00	–

*1. First week pre-extension = First week of quarantine before extension, including participants answering during 17–23 March 2020; 2. Second week pre-extension = Second week of quarantine before extension, including participants answering during 24–29 March 2020; 3. First extension = Sub-period after the first quarantine extension, including participants answering during 30 March to 10 April 2020; 4. Second/third extensions = Sub-period after the second quarantine extension and including the third extension, with participants answering during 11 April to 08 May 2020; 5. Fourth extension = Sub-period after the fourth quarantine extension, including participants answering during 09–23 May 2020; 6. Fifth extension = Sub-period after the fifth quarantine extension, including participants answering during 24 May to 04 June 2020*.

a *Pairwise comparisons of proportions were carried out with Test of equal or given proportions, alternative hypothesis two-sided*.

b *Exact p-values are informed, except for p-values under 0.001, which are informed as <0.001. Statistically significant p-values are highlighted in bold*.

### Regression Models for General Mental Health State Indicators

The initial regression model for each general MHS indicator included the predictors: age, sites of residence by prevalence of COVID-19 cases, mental disorder history, suicide attempt history, and sub-periods of quarantine duration. The minimum suitable model best fitting the data was the same as the model from the start, i.e., included all the predictors, for the MHS indicators self-perceived health [F_(11 and 5001)_ = 52.44, *p* < 0.001, Residuals: −7.88 to 9.12; AIC = 11941.75], psychological discomfort [F_(11 and 5001)_ = 31.87, *p* < 0.001, Residuals: −4.39 to 4.13; AIC = 5911.25], and social functioning and coping [F_(11 and 5001)_ = 56.49, *p* < 0.001, Residuals: −3.63 to 5.45; AIC = 6248.35; Table 6]. This model explained 10.34% of variance in the participants' self-perceived health according to *r*^2^ (10.14% according to adjusted *r*^2^), with a RSE of 3.29, corresponding to 57.58% error rate. For psychological discomfort, the model explained only 6.55% of variance according to *r*^2^ (6.34% according to adjusted *r*^2^), with a RSE of 1.80, corresponding to 53.30% error rate. For both self-perceived health and psychological discomfort, the largest effect sizes corresponded to the predictors: suicide attempt history and sub-periods of quarantine duration (Table 7). For social functioning and coping, the model explained 11.05% of variance according to *r*^2^ (10.86% according to adjusted *r*^2^), with a RSE of 1.86, corresponding to 80% error rate. The largest effect sizes corresponded to the predictors: suicide attempt history and age (Table 7). Overall, being a younger age, having mental disorder history, and longer quarantine durations were correlated to worst self-perceived health, higher levels of psychological discomfort, and worst social functioning and coping; while lack of previous suicide attempt and residing in sites with intermediate prevalence of COVID-19 cases had a protective effect on these MHS indicators. Residing in sites with a low prevalence of COVID-19 also had a protective effect on psychological discomfort (Table 6).

**Table 6:** Summary of the linear regression models better fitting^a^ each general mental health state (MHS) indicator (*N* = 5,013).

**MHS indicator**	**Predictors**	**Estimate**	**Std. Error**	***t*-value**	***p-value* ^b^**	**95% CI**	
						**2.5%**	**97.5%**
Self-perceived health	Intercept	7.63	0.16	46.19	**< 0.001**	7.31	7.96
	Age	−0.07	0.01	−12.00	**< 0.001**	−0.08	−0.06
	Prevalence of COVID-19: intermediate	−0.31	0.10	−2.98	**0.003**	−0.51	−0.11
	Prevalence of COVID-19: low	−0.21	0.13	−1.57	0.12	−0.47	0.05
	Mental disorder history: yes	0.31	0.11	2.87	**0.004**	0.10	0.53
	Suicide attempt history: no	−1.51	0.10	−14.63	**< 0.001**	−1.71	−1.30
	Suicide attempt history: yes	−0.13	0.18	−0.70	0.48	−0.48	0.23
	Quarantine sub-periods: 2. 2nd pre-ext.	0.19	0.17	1.09	0.28	−0.15	0.52
	Quarantine sub-periods: 3. 1st ext.	0.64	0.15	4.30	**< 0.001**	0.35	0.93
	Quarantine sub-periods: 4. 2nd/3rd ext.	1.45	0.16	8.82	**< 0.001**	1.13	1.77
	Quarantine sub-periods: 5. 4th ext.	1.33	0.16	8.16	**< 0.001**	1.01	1.65
	Quarantine sub-periods: 6. 5th ext.	1.46	0.14	10.63	**< 0.001**	1.19	1.73
Psychological discomfort	Intercept	4.10	0.09	45.26	**< 0.001**	3.92	4.28
	Age	−0.03	0.003	−8.66	**< 0.001**	−0.03	−0.02
	Prevalence of COVID-19: intermediate	−0.19	0.06	−3.27	**0.001**	−0.30	−0.07
	Prevalence of COVID-19: low	−0.14	0.07	−1.96	**0.05**	−0.29	0.0003
	Mental disorder history: yes	0.17	0.06	2.90	**0.004**	0.06	0.29
	Suicide attempt history: no	−0.57	0.06	−10.04	**< 0.001**	−0.68	−0.46
	Suicide attempt history: yes	−0.14	0.10	−1.40	0.16	−0.33	0.05
	Quarantine sub-periods: 2. 2nd pre-ext.	−0.01	0.09	−0.06	0.95	−0.19	0.18
	Quarantine sub-periods: 3. 1st ext.	0.22	0.08	2.73	**0.007**	0.06	0.38
	Quarantine sub-periods: 4. 2nd/3rd ext.	0.72	0.09	8.01	**< 0.001**	0.54	0.90
	Quarantine sub-periods: 5. 4th ext.	0.67	0.09	7.54	**< 0.001**	0.50	0.85
	Quarantine sub-periods: 6. 5th ext.	0.73	0.07	9.75	**< 0.001**	0.59	0.88
Social functioning and coping	Intercept	3.53	0.09	37.73	**< 0.001**	3.35	3.72
	Age	−0.04	0.003	−12.78	**< 0.001**	−0.05	−0.03
	Prevalence of COVID-19: intermediate	−0.12	0.06	−2.09	**0.04**	−0.24	−0.01
	Prevalence of COVID-19: low	−0.07	0.08	−0.88	0.38	−0.22	0.08
	Mental disorder history: yes	0.14	0.06	2.26	**0.02**	0.02	0.26
	Suicide attempt history: no	−0.94	0.06	−16.11	**< 0.001**	−1.05	−0.83
	Suicide attempt history: yes	0.01	0.10	0.11	0.91	−0.19	0.21
	Quarantine sub-periods: 2. 2nd pre-ext.	0.19	0.10	1.98	**0.05**	0.002	0.38
	Quarantine sub-periods: 3. 1st ext.	0.42	0.08	4.95	**< 0.001**	0.25	0.58
	Quarantine sub-periods: 4. 2nd/3rd ext.	0.73	0.09	7.82	**< 0.001**	0.55	0.91
	Quarantine sub-periods: 5. 4th ext.	0.66	0.09	7.11	**< 0.001**	0.47	0.84
	Quarantine sub-periods: 6. 5th ext.	0.73	0.08	9.34	**< 0.001**	0.58	0.88
Psychological distress	Intercept	31.99	0.36	89.11	**< 0.001**	31.29	32.70
	Age	−0.18	0.02	−13.90	**< 0.001**	−0.20	−0.15
	Mental disorder history: yes	2.32	0.25	9.34	**< 0.001**	1.83	2.80
	Suicide attempt history: no	−5.09	0.23	−21.80	**< 0.001**	−5.54	−4.63
	Suicide attempt history: yes	1.31	0.41	3.20	**0.001**	0.51	2.12
	Quarantine sub-periods: 2. 2nd pre-ext.	0.13	0.39	0.34	0.73	−0.63	0.89
	Quarantine sub-periods: 3. 1st ext.	0.16	0.34	0.49	0.62	−0.50	0.83
	Quarantine sub-periods: 4. 2nd/3rd ext.	2.22	0.37	6.02	**< 0.001**	1.50	2.95
	Quarantine sub-periods: 5. 4th ext.	2.11	0.35	5.97	**< 0.001**	1.42	2.80
	Quarantine sub-periods: 6. 5th ext.	2.63	0.31	8.44	**< 0.001**	2.02	3.24

*Std. Error, Standard error; 95% CI, 95% Confidence Interval; Prevalence of COVID-19 (Sites of residence by prevalence of COVID-19 cases): Low, up to 10 confirmed cases of COVID-19 per 100,000 inhabitants; Intermediate, between 11 and 25 confirmed cases of COVID-19 per 100,000 inhabitants; High, > 50 confirmed cases of COVID-19 per 100,000 inhabitants; Mental disorder history: yes, Presence of mental disorder history; no, Absence of mental disorder history; Suicide attempt history: yes, Presence of suicide attempt history; no, Absence of suicide attempt history; Quarantine sub-periods: 1. 1st pre-ext., First week of quarantine before extension, including participants answering during 17–23 March 2020; 2. 2nd pre-ext., Second week of quarantine before extension, including participants answering during 24–29 March 2020; 3. 1st ext., Sub-period after the first quarantine extension, including participants answering during 30 March to 10 April 2020; 4. 2nd/3rd ext., Sub-period after the second quarantine extension and including the third extension, with participants answering during 11 April to 08 May 2020; 5. 4th ext., Sub-period after the fourth quarantine extension, including participants answering during 09–23 May 2020; 6. 5th ext., Sub-period after the fifth quarantine extension, including participants answering during 24 May to 04 June 2020*.

a *Best fitted model according to multiple linear regressions: stepwise selection (direction: both) by using the exact Akaike's Information Criterion (AIC) to compare additive fitted models*.

b *Exact p-values are informed, except for p-values under 0.001, which are informed as <0.001. Statistically significant p-values are highlighted in bold*.

**Table 7:** Summary of Cohen's effect size for models better fitting each general mental health state (MHS) indicator (*N* = 5,013).

**MHS indicator**	**Predictors**	***f***	**90% CI**	
Self-perceived health	Age	0.16	0.14	0.19
	Cases per inhabitant	0.05	0.02	0.07
	Mental disorder history	0.09	0.07	0.12
	Suicide attempt history	0.21	0.18	0.23
	Sub-periods of quarantine duration	0.19	0.16	0.21
Psychological discomfort	Age	0.11	0.08	0.13
	Cases per inhabitant	0.05	0.02	0.07
	Mental disorder history	0.08	0.05	0.10
	Suicide attempt history	0.14	0.11	0.16
	Sub-periods of quarantine duration	0.18	0.15	0.20
Social functioning and coping	Age	0.19	0.16	0.21
	Cases per inhabitant	0.04	0.01	0.06
	Mental disorder history	0.09	0.07	0.12
	Suicide attempt history	0.23	0.21	0.26
	Sub-periods of quarantine duration	0.16	0.13	0.18
Psychological distress	Age	0.22	0.20	0.24
	Mental disorder history	0.23	0.20	0.28
	Suicide attempt history	0.34	0.31	0.36
	Sub-periods of quarantine duration	0.15	0.13	0.17

*f, Cohen's f (partial); 90% CI, 90% Confidence Interval*.

For psychological distress, the best fitting model included almost all of the predictors as the model from the start, except sites of residence by prevalence of COVID-19 cases [F_(9 and 5003)_ = 132.10, *p* < 0.001, Residuals: −22.87 to 25.67; AIC = 20143.82; Table 6]. This model explained 19.20% of variance in the participants' psychological distress according to *r*^2^ (19.06% according to adjusted *r*^2^) with a RSE of 7.45, corresponding to 28.25% error rate. Being a younger age, having mental disorder history, having suicide attempt history, and longer quarantine durations were correlated to higher levels of psychological distress; while lack of previous suicide attempt had a protective effect on this MHS indicator. The largest effect sizes corresponded to the predictors: suicide attempt history and mental disorder history (Table 7).

## Discussion

### Differences in General Mental Health State by Sites of Residence With Different Prevalence of COVID-19

In the first aim of this research, we analyzed differences in general MHS indicators, in Argentinean women, by sites of residence with different prevalence of COVID-19 cases. Worse self-perceived health and higher psychological discomfort affected significantly more women residing in sites with high prevalence of COVID-19 cases, compared to those residing in sites with intermediate prevalence of this disease. At a first glance, it could be presumed that mental health impacts on women during quarantine may be attributed to the objective risk of contagion (greater or less measured in an area). However, our findings do not support this assumption due to a number of reasons. First, these MHS indicators are worse in sites with low prevalence of COVID-19 compared to sites with intermediate prevalence of this disease. Second, rather than differences are meaningful between sites with high and low prevalence of COVID-19, mean scores are equal (in self-perceived health) or quite similar (in psychological discomfort) between these sites. Third, the remaining MHS indicators, i.e., social functioning and coping and psychological distress, do not differ by sites of residence with low, intermediate, and high prevalence of COVID-19. Fourth, when statistical significant differences were found, effect size measures were very small. Fifth, in sites of residence with low as well as with intermediate and high prevalence of COVID-19, mean scores of self-perceived health and of psychological distress overcome the cutoff scores for mental disorders. Some of these findings are consistent with what we have previously found on college students, whose differences in psychological discomfort by regions of residence were only meaningful between the most populated and center regions, which correspond to sites with high and intermediate prevalence of COVID-19 cases, respectively, but not between the remaining sites of residence (López Steinmetz et al. under review). Unlike COVID-19 cases, mental health affections seem to be equally distributed throughout the whole country, which may suggest that the prevalence of the latter may be higher than the former. In line with our findings, a study carried out in China found that the specific location of residence, within or outside the epicenter of the pandemic, do not seem to be significantly associated to more or less mental health problems; instead of the specific location, the direct exposure to COVID-19 seems to be relevant (24).

### Differences in General Mental Health State by Quarantine Sub-periods

Regarding the second aim of this research, the mean scores of all general MHS indicators in women are significantly worse for longer quarantine sub-periods (up to 53, 68, and 80-day duration, i.e., second/third, fourth, and fifth extensions, respectively) than for shorter sub-periods (up to seven, 13, and 25-day duration, i.e., first and second week of pre-quarantine extension, and first extension, respectively), and these differences are somewhat largest for self-perceived health and psychological discomfort than for social functioning and coping and psychological distress. This worsening pattern that we have found on mental health as time went by does not seem to be privative of women, since we have also observed it in college students (López Steinmetz et al. under review) and in the general population of both sexes (López Steinmetz et al. under review). Although this worsening pattern is not observed solely in women, the possibility exists that negative mental health impact may be worse in women than in men (25, 26). In line with our results, a current study has also found that mental health state worsens as the time spent in lockdown has progressed (27). On the contrary, findings of a study carried out in China by Wang et al. (26) reported a significant reduction on post-traumatic stress disorder symptoms along with no significant longitudinal changes in stress, anxiety, and depression 4 weeks after the COVID-19 outbreak. Unfortunately, in the Chinese study, the authors did not indicate if both measures—during the initial outbreak and 4 weeks later—or just one of them was under social isolation sanitary measures. In addition, it is important to note that the results of the cited study was conducted prior to the COVID-19 infection reaching the state of pandemic and although it is announced as a longitudinal study, the majority of data (1,405 participants of *N* = 1,738) analyzed in such study are in fact transversal samples, as our samples are. Our results are in line with findings based on previous quarantine-related situations reporting that longer durations of quarantine are associated with increased psychological symptoms (28–30). However, it is important to note that most of these previous studies investigating the impact of quarantine duration focused on people quarantined because they became infected by a particular disease, or belong to particular occupational groups, such as nurses and other healthcare workers. However, it was demonstrated that healthcare workers tend to be at high risk of developing mental illness than other occupational groups during current (31) and previous (32) epidemics and pandemics. Bearing all these in mind, our findings are novel since they bring additional insights on mental health impact of quarantine duration in non-infected women of the general population.

Finally, in the additional exploratory analyses corresponding to aim 2, we noticed some differences in the composition of participants' sub-group regarding with age—where greater mean age of sub-groups corresponded to longer quarantine durations—and also regarding suicide attempt history—where greater proportions of participants without suicide attempt history and lower proportions of participants with suicidal ideation history responded during longer quarantine durations. On the one hand, these results indicate that, since we have not included covariates in ANOVAs, differences on MHS by quarantine durations and further analyses (i.e., multiple linear regressions) should be interpreted with caution. Although this is true, on the other hand, all these results indirectly indicate that when worse mean scores on MHS were recorded (i.e., during longer quarantine durations): (a) older participants were responding to the survey, and (b) higher proportions of participants without suicide attempt history and lower proportions of participants with suicidal ideation history were responding to the survey. Thus, if younger age results as being a predictor of worse MHS and if the absence of suicide attempt history results as being a protective factor for MHS this would not be due to differences in the composition of participants' sub-group.

### Regression Models for General Mental Health State Indicators

When assessing the effects on each specific MHS indicator of potentially affecting factors, we found that—in general terms—being a younger age, having mental disorder history, and longer quarantine durations are associated to worsening mental health in women, while a lack of previous suicide attempt has a protective effect on all the MHS indicators measured. Residing in sites with intermediate prevalence of COVID-19 cases also provided a protective effect for self-perceived health, psychological discomfort, and social functioning and coping in women. Regarding age, prior to the current pandemic, literature reported that young people were one of the most vulnerable age groups for developing mental health disorders (33, 34). With the current mass quarantine for the COVID-19 pandemic, schools and college closures have been conducted in hundreds of countries, such as Argentina, affecting more females than males (35). Such closures along with other activity cessations (e.g., group sports activities) disallow young people to access social support organizations e.g., peer support groups, and may in turn cause additional negative mental health impacts (36), thus increasing the vulnerability in developing mental disorders. In line with our findings, a younger age and female gender were previously indicated as pre-quarantine predictors associated with negative psychological impacts (6, 37).

It is important to note that school closures not only disrupt the lives of students, but also of their families (38). This would mean a challenge for parents, who must acquire and perform additional functions, such as emerging educators. Pandemic-related sanitary measures like quarantine, place parents as the first-line of responders for children's survival, care, and learning (39). However, in Argentina these additional roles are overloaded mainly to women, who became full-time caregivers, are over-worked, experienced more fatigue than before the quarantine, and sleep less than necessary (40). It may be presumed that this is the case not only in Argentina. In non-quarantine-related situations, women still undertake twice as much routine housework as men do, and more unbalanced divisions of housework were associated to greater depression and less marital satisfaction in women (41). However, across the transition to parenthood, for working-class women, the division of child-care could be more relevant in predicting distress than the division of housework (42). Nonetheless, there are evidences to expect that increased family demands are likely to be primarily shouldered by women (43). In addition, quarantine situations have the potential of exacerbating intimate partner violence, which is most frequently suffered by women than by men (44, 45). Women who have undergone intimate partner violence are, in turn, at a greater risk of multiple mental health and physical conditions (46).

### Conclusions

All the aspects described above suggest that women are a special vulnerable group for developing mental disorders during quarantine. As longer quarantine durations (6, 28–30) and its extensions (47) were demonstrated as having a negative impact on mental health, and some of these effects may be long-lasting (48), there is an imperative need that the Government provides funding sources for developing sanitary programs targeted at the mental health of women. Our results suggest that special attention needs to be paid to younger women and also to women having a history of mental disorder. Along with physical health, mental health and psychological needs must start to be a priority for the Government during and after quarantine and the COVID-19 pandemic. In sanitary events such as epidemics and pandemics, the amount of people resulting with mental health affections is usually higher than people affected by the physical disease, and negative mental health effects tend to persist longer than the epidemic or pandemic; however, mental health or psychological needs have never been a priority during this kind of sanitary events (49, 50). As having a worsening mental health was demonstrated as being an adverse effect of pandemic-related sanitary measures (7, 11), which affects more in groups at particular risk, such as women (11), and although these negative mental health outcomes may not be entirely prevented, it should be addressed early. In this regard, findings of our study may be useful for public health officials and government officials who must decide upon sanitary measures and public policies; however, they need to be interpreted with caution and considered within the context of several limitations. First, this study was cross-sectional. However, we implemented successive sampling, which allowed us to monitor group central tendency measures through quarantine sub-periods; although prospective research is warranted. Second, our sample was one of convenience and it is unclear to what extent our results could be representative of the Argentinean women population. Nevertheless, it is important to note that we have analyzed a large sample (> 5,000), including data from participants throughout the whole country. Third, the sample was limited to a single country, thus, findings can only be interpreted within the context of Argentinean women. Fourth, our sample only includes women having access to the internet; thus, low-income women may be underrepresented. Likewise, we did not capture additional relevant factors such as family/household demands and domestic violence, which should be addressed in further research. Fifth, mental disorder was assessed as a binary variable, which does not adequately describe the complexity of mental health in the population, and the screening tools used provide limited information. Sixth, additional variables, such as physical comorbidities, pregnancy and postpartum conditions, should be included in further research, since these might have an influence on general mental health state. Despite these limitations, we believe that our findings remain valuable for developing evidence-based sanitary measures and help shed light for further research on women mental health impacts during the current quarantine, which is a pressing public health concern.

## Data Availability Statement

All datasets generated for this study are included in the article/Supplementary Material.

## Ethics Statement

This study involved human participants and was reviewed and approved by Ethics Committee of the Institute of Psychological Research, Faculty of Psychology, National University of Córdoba (CEIIPsi-UNC-CONICET; comite.etica.iipsi@psicologia.unc.edu.ar">comite.etica.iipsi@psicologia.unc.edu.ar). The participants provided their written informed consent to participate in this study.

## Author Contributions

LL has elaborated the research project, designed the online protocol of this research, participated in the data collection, made data analyzes, and wrote the manuscript. SF has participated in the data collection, made bibliography searches, and revised the manuscript for English grammar. CL and MD have participated in the data collection and carried-out bibliography searches. JG has participated in the data collection and revised the manuscript. All authors contributed to the article and approved the submitted version.

## Conflict of Interest

The authors declare that the research was conducted in the absence of any commercial or financial relationships that could be construed as a potential conflict of interest.
