# Supplementary material for: General Mental Health State Indicators in Argentinean Women During Quarantine of up to 80-Day Duration for COVID-19 Pandemic
Source: Front Glob Womens Health. 2020 Sep 17;1:580652. doi: 10.3389/fgwh.2020.580652 (PMC8593979; doi:10.3389/fgwh.2020.580652)
Supplement: Supplementary file 2 [file Data_Sheet_2.docx]

**Supplementary materials for *General mental health state indicators in Argentinean women during quarantine of up to 80-day duration for COVID-19 pandemic***

Lorena Cecilia López Steinmetz, Shao Bing Fong, Candela Abigail Leyes, María Agustina Dutto Florio, Juan Carlos Godoy

| **Table S1.** Distribution of the sample according to site of residence. | | |
| --- | --- | --- |
| **Site of residence** | **Participants** | |
|  | **f** | **%** |
| Jujuy | 243 | 4.85 |
| Salta | 199 | 3.97 |
| Tucumán | 27 | 0.54 |
| Catamarca | 8 | 0.16 |
| La Rioja | 8 | 0.16 |
| Santiago del Estero | 15 | 0.30 |
| Formosa | 7 | 0.14 |
| Chaco | 16 | 0.32 |
| Misiones | 18 | 0.36 |
| Corrientes | 9 | 0.18 |
| Entre Ríos | 26 | 0.52 |
| Santa Fe | 335 | 6.68 |
| Córdoba | 1896 | 37.82 |
| Buenos Aires Province | 1427 | 28.47 |
| Buenos Aires City (CABA) | 566 | 11.29 |
| San Luis | 9 | 0.18 |
| San Juan | 6 | 0.12 |
| Mendoza | 25 | 0.50 |
| La Pampa | 14 | 0.28 |
| Neuquén | 16 | 0.32 |
| Río Negro | 25 | 0.50 |
| Chubut | 11 | 0.22 |
| Santa Cruz | 18 | 0.36 |
| Tierra del Fuego | 67 | 1.34 |
| Abroad | 22 | 0.44 |
| Total | 5013 | 100 |
| *Notes*: Abroad = Argentinean participants currently stranded abroad due to travel bans and airport closures for the COVID-19 pandemic. | | |

**Skewness and kurtosis in each indicator measured of the general mental health state**

*Self-perceived health*:

Skewness = 0.12

Kurtosis = 1.96

*Psychological discomfort*:

Skewness = -0.23

Kurtosis = 1.96

*Social functioning and coping*:

Skewness = 0.42

Kurtosis = 1.93

*Psychological distress*:

Skewness = 0.19

Kurtosis = 2.26

| **Table S2.** Quantity of confirmed cases of COVID-19 per 100,000 inhabitants up to 10^th^ June 2020^a^ by site of residence | |
| --- | --- |
| **Provinces** | **Cases x 100,000 inhabitants** |
| Jujuy | 1 |
| Salta | 1 |
| Tucumán | 2 |
| Catamarca | n.d. |
| La Rioja | 16 |
| Santiago del Estero | 2 |
| Formosa | n.d. |
| Chaco | 92 |
| Misiones | 2 |
| Corrientes | 8 |
| Entre Ríos | 4 |
| Santa Fe | 7 |
| Córdoba | 12 |
| Buenos Aires Province | 54 |
| Buenos Aires City (CABA) | 388 |
| San Luis | 2 |
| San Juan | 0 |
| Mendoza | 5 |
| La Pampa | 1 |
| Neuquén | 23 |
| Río Negro | 65 |
| Chubut | 4 |
| Santa Cruz | 13 |
| Tierra del Fuego | 78 |
| *Notes*: n.d. = no data.  ^a^ Information based on available official data published by the Argentinean Government on 10^th^ June 2020 (22). | |

| **Table S3.** Multicollinearity assessment by using the VIF, the tolerance statistics, and the mean VIF, with the *VIF* function from the *car* package of R | | | |
| --- | --- | --- | --- |
| **The VIF** | | | |
| **Predictors** | **GVIF** | **Df** | **GVIF ^(1/(2*Df))** |
| Age | 1.13 | 1 | 1.06 |
| Sites of residence by prevalence of COVID-19 cases | 1.14 | 2 | 1.03 |
| Mental disorder history | 1.09 | 1 | 1.04 |
| Suicide attempt history | 1.12 | 2 | 1.03 |
| Quarantine duration | 1.24 | 5 | 1.02 |
| **The tolerance statistics** | | | |
| **Predictors** | **GVIF** | **Df** | **GVIF ^(1/(2*Df))** |
| Age | 0.89 | 1.0 | 0.94 |
| Sites of residence by prevalence of COVID-19 cases | 0.88 | 0.5 | 0.97 |
| Mental disorder history | 0.92 | 1.0 | 0.96 |
| Suicide attempt history | 0.89 | 0.5 | 0.97 |
| Quarantine duration | 0.80 | 0.2 | 0.98 |
| **The mean VIF** | | | |
| 1.46 | | | |
